# A Tale of Four Stories: Soil Ecology, Theory, Evolution and the Publication System

**DOI:** 10.1371/journal.pone.0001248

**Published:** 2007-11-28

**Authors:** Sébastien Barot, Manuel Blouin, Sébastien Fontaine, Pascal Jouquet, Jean-Christophe Lata, Jérôme Mathieu

**Affiliations:** 1 UMR 137, Institut de Recherche pour le Développement (IRD), Bondy, France; 2 INRA, Unité d'agronomie, Clermont Ferrand, France; 3 UMR 8079, Université Paris XI, Orsay, France; 4 UMR 137, Université Paris VI, Bondy, France; University of Helsinki, Finland

## Abstract

**Background:**

Soil ecology has produced a huge corpus of results on relations between soil organisms, ecosystem processes controlled by these organisms and links between belowground and aboveground processes. However, some soil scientists think that soil ecology is short of modelling and evolutionary approaches and has developed too independently from general ecology. We have tested quantitatively these hypotheses through a bibliographic study (about 23000 articles) comparing soil ecology journals, generalist ecology journals, evolutionary ecology journals and theoretical ecology journals.

**Findings:**

We have shown that soil ecology is not well represented in generalist ecology journals and that soil ecologists poorly use modelling and evolutionary approaches. Moreover, the articles published by a typical soil ecology journal (Soil Biology and Biochemistry) are cited by and cite low percentages of articles published in generalist ecology journals, evolutionary ecology journals and theoretical ecology journals.

**Conclusion:**

This confirms our hypotheses and suggests that soil ecology would benefit from an effort towards modelling and evolutionary approaches. This effort should promote the building of a general conceptual framework for soil ecology and bridges between soil ecology and general ecology. We give some historical reasons for the parsimonious use of modelling and evolutionary approaches by soil ecologists. We finally suggest that a publication system that classifies journals according to their Impact Factors and their level of generality is probably inadequate to integrate “particularity” (empirical observations) and “generality” (general theories), which is the goal of all natural sciences. Such a system might also be particularly detrimental to the development of a science such as ecology that is intrinsically multidisciplinary.

## Introduction

Soils constitute a primordial compartment of terrestrial ecosystems. They are the interface between earth mineral layer and the biosphere. They result both from the degradation of the mineral parent rock releasing essential nutrients for life, and the accumulation of dead organic matter. Nutrients sequestrated in dead organic matter are recycled by soil microbes, which is an essential condition for the maintenance of primary production. Moreover, huge quantities of carbon are sequestered in the recalcitrant part of soil organic matter over centuries to millennia [1], [2] before being released as CO_2_. On the long term, this sequestration influences the quantity of atmospheric CO_2_ and the climate [3]. Hence, soils play a fundamental role in all biogeochemical cycles.

Soil processes depend on physical and chemical parameters (climate, parent rock) but also depend on many soil organisms such as bacteria, fungus, mesofauna (collembola, mite), macrofauna (earthworm, termite, ant, insect larvae, millipede…) and plants. The study of the interactions between these organisms, and between these organisms and their physical environment has required the development of a whole scientific domain: soil ecology. Besides the desire to increase ecological knowledge, the importance of the involved applied issues (soil fertility, soils as a carbon sink…etc) has strongly fostered the development of soil ecology as proved by the existence of many specialized journals. The field is currently making wide progresses. For example, many recent studies reveal new mechanisms that could deeply influence soil fertility, competition between plants or ecosystem reaction to global change [4]–[6]. However, the feeling has developed among some soil scientists that soil ecology has developed too independently from the rest of ecology and that soil ecology is short of modelling and evolutionary approaches [7]–[9]. Hence soil ecology seems to have had a small influence on the development of contemporary ecology and, conversely, many useful ecological concepts have not been used to interpret soil processes.

To test quantitatively the veracity of these hypotheses and to analyse better the place of soil ecology within ecology we have achieved a wide bibliographic analysis. We aimed at providing the evidence that soil ecologists make a parsimonious use of modelling and evolutionary interpretations: (1) soil ecology journals publish low percentages of articles based on a modelling or evolutionary approaches, (2) journals specialized in evolution and modelling publish low percentages of articles related to soil ecology and (3) there are low percentages of cross-citations between soil ecology journals and journals specialized in modelling and evolutionary approaches. We also aimed at showing that the links between soil ecology and general ecology are sparse: (4) there are low percentages of soil ecology articles published in generalist ecology journals and (5) there are low percentages of cross-citations between generalist ecology journals and soil ecology journals. We thus tested five hypotheses. In the discussion we analysed the results of these five tests and try to give proximal and historical explanations to the position of soil ecology within ecology. We finally conclude with some remarks on the way the publication system may have influenced the development of soil ecology.

## Methods

To assess the position of soil ecology within general ecology and to evaluate the use of modelling and evolutionary approaches we have analysed eight journals specialized in soil ecology (*Applied Soil Ecology*, *Biology and Fertility of Soils*, *European Journal of Soil Biology*, *Pedobiologia*, *Soil Biology and Biochemistry*, *Plant and Soil*), seven generalist journals of ecology (*American Naturalist*, *Ecology*, *Ecology Letters*, *J. Animal Ecology*, *J. Ecology*, *Functional Ecology*, *Oikos*), two journals specialized in evolution (*Evolution and Evolutionary Ecology*), and three journals specialized in modelling (*Ecological Modelling*, *Theoretical Population Biology*, *Journal of Theoretical Biology*). *J. Animal Ecology*, *J. Ecology* and *Functional Ecology* are arguably less general than the other generalist journals but taken together they are the equivalent of Ecology for the British Ecological Society. This list is not comprehensive but was designed to allow comparing clear-cut cases, i.e. journals publishing respectively only articles related to soil ecology, evolution and modelling and journals publishing articles related to all fields of ecology.

The data base, ISI Web of Knowledge, was scanned from 1997 to 2004 (from 1998 for *Ecology Letters*). Overall, this represents about 23000 papers for the considered journals. To find all articles dealing with soil ecology in journals not specialized in soil ecology the word “soil” was searched for in titles, key-words and abstracts. Conversely, it was searched for the words “evolution” and “model” in journals not specialized in evolution or modelling. All abstracts were looked through to eliminate irrelevant articles. We checked beforehand that searching for these general terms allows gathering most relevant papers but that the “manual” sorting was necessary due to the poor selectivity of these terms. It is difficult to give a precise definition of soil ecology. However, we considered as linked to soil ecology any study dealing with soil organisms, parts of organisms dwelling in soils (root), soil processes involving organisms (mineralization, soil respiration) or processes linking soil and aboveground organisms. For the selection of papers dealing with models, studies only using statistical models or null models were rejected as well as studies only mentioning a published model to state that their empirical results support or not the conclusions of these models. For the selection of papers dealing with evolution, studies addressing directly an evolutionary issue or only interpreting empirical data using evolutionary theories were taken into account.

We first calculated, for each journal, the percentage of articles dealing with soil ecology, modelling and evolution. We then assessed the relations between the percentage of articles dealing with soil ecology and respectively the Impact Factor of the journals, the percentage of articles based on modelling and the percentage of articles based on an evolutionary approach. This allowed comparing journals and types of journals (specialized in soil ecology vs. generalist or specialized in evolution or modelling) but this did not allow comparing articles published in different fields of ecology (for example bird ecology vs. soil ecology). To approach the latter we have also calculated, inside the publications of each of our sample of twelve journals that are non-specialized in soil ecology, the percentage of articles dealing with soil ecology that also use models or also involve evolutionary interpretations. Then, these percentages were compared (χ^2^ test) to the percentages of publications, inside the same non-specialized journals, using models or evolutionary interpretations but not dealing with soil ecology, i.e. publications related to all other fields of ecology such as bird ecology.

The development of a scientific field should also be reflected in the publications of very generalist and highly cited journals such as Science and Nature. We searched for the articles dealing with soils published in these journals between 1997 and 2004 (searching for the word soil in the title, abstract and key-words and eliminating manually non relevant articles) and classified coarsely the content of these articles.

So far, the analyses aimed at assessing the position of soil ecology within general ecology by counts of articles dealing with soil ecology in different categories of journals. Citations might also constitute important links between scientific fields and more specifically between soil ecology and general ecology/theory/evolutionary thinking. We have thus examined the articles cited by the articles of three issues of *Soil Biology and Biochemistry* (2003, volume 35, issues 10, 11, 12) and the articles citing these articles published in SBB. These articles were classified in broad categories: Model, Generalist journals, Animal, Plant, Ecology, Agronomy, Soil sciences, Microbiology, Miscellaneous, Soil Ecology. “Model” refers to the journal specialized in modelling (the one cited above in the first paragraphs of the section). “Generalist journals” are journals such as *Science*, *Nature* and *Proceedings of the Royal Society London*. “Animal” and “Plant” refer to journals studying animals and plant but not specifically their ecology (for example Nematology and Plant Physiology). “Ecology” and “Soil ecology” refers to generalist ecology journals and journals specialized in soil ecology such as the once cited above in the first paragraph of the section. “Agronomy” refers to journals specialized in the application of soil and ecological sciences for plant production. “Soil sciences” refer to journals about soils but with little emphasis on biological and ecological processes such as the European Journal of Soil Sciences. “Microbiology” refers to microbiology journals. “Miscellaneous” refers to journals difficult to classify, mostly journals about specific scientific tools such as *Rapid Communications in Mass Spectrometry* or journals about specific type of environment such as *Canadian Journal of Forest Research*.

## Results

**Figure 1:**
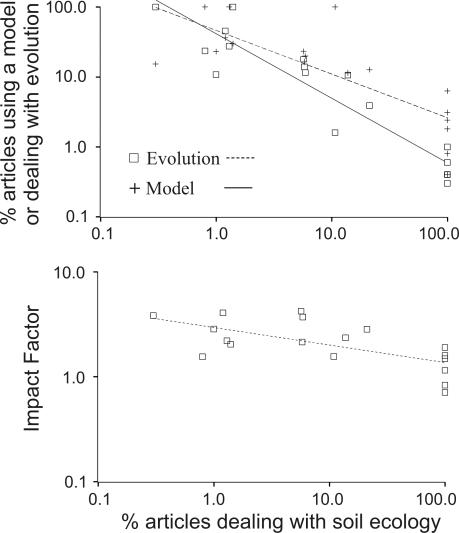
Use of models and evolutionary thinking in ecology journals and link with Impact Factors. Top panel, relation between the percentage of articles dealing with soil ecology and (1) the percentage of articles using a model, R^2^ = 0.62, F = 25.8, P<0.0001; (2) the percentage of articles dealing with evolution, R^2^ = 0.87, F = 110.5, P<0.0001. Bottom panel, relation between the percentage of articles dealing with soil ecology and the Impact Factor, R^2^ = 0.43, F = 12.0, P = 0.0032. Each point corresponds to one of the eighteen journals investigated. Axes have a logarithmic scale. The log-log linear regression is highly significant for each of these relations with a negative slope in each case. For each relation both the raw data and the regression line are displayed. See Table 1 for raw data.

As expected, Journals specialized in soil ecology have lower impact factors (IF) than generalist journals (Table 1, see also Fig. 1 the log-log significant relation between IF and percentages of papers dealing with soil ecology). More interestingly, a low percentage of the papers published in the generalist journals deals with soil ecology; less than 6 % in most cases but 20% for *Journal of Ecology* and 13.8% for *Oikos*.

**Table 1:** Summary of the bibliographical analysis (see text for details).

	Total numberof articles	Total soil	Model			Evolution			IF 2003
			Total	Within non soil	Within soil	Total	Within non soil	Within soil	
*Appl. Soil Ecol.*	626	100.0	2.4	.	2.4	0.3	.	0.3	1.48
*Biol. Fert. Soils*	586	100.0	1.8	.	1.8	0.1	.	0.1	1.15
*Eur. J Soil Biol.*	239	100.0	0.4	.	0.4	0.4	.	0.4	0.83
*Pedobiologia*	1843	100.0	0.8	.	0.8	1.0	.	1.0	0.71
*Plant Soil*	1121	100.0	6.3	.	6.3	0.6	.	0.6	1.59
*Soil Biol. Biochem.*	2337	100.0	3.1	.	3.1	0.4	.	0.4	1.90
*Am. Nat.*	1072	1.2	36.2	35.8	69.2*	45.6	**46.0**	15.4*	4.06
*Ecology*	2279	5.9	19.9	**20.5**	10.5*	11.5	**11.9**	5.2*	3.70
*Ecol. Lett.*	671	5.7	23.2	**24.2**	7.9*	17.9	**18.5**	7.9*	4.21
*J. Animal Ecol.*	779	1.0	23.0	**23.1**	**12.5**	10.8	10.6	25.0	2.84
*J. Ecol.*	716	21.2	12.7	**13.5**	9.9*	3.9	3.4	5.9*	2.83
*Func. Ecol.*	1975	5.8	16.4	**16.6**	13.2*	13.9	**10.6**	7.9*	2.14
*Oikos*	861	13.8	11.4	**15.4**	5.9*	10.6	**14.3**	10.1*	2.35
*Evolution*	1845	0.3	15.3	**29.4**	**0.0**	100.0	100.0	100.0	3.83
*Evol. Ecol.*	352	1.4	29.8	20.2	60.0	100.0	100.0	100.0	2.04
*Ecol. Model.*	1799	10.7	100.0	100.0	100.0	1.6	**1.7**	1.0*	1.56
*Theor. Pop. Biol.*	445	1.3	100.0	100.0	100.0	27.6	**28.0**	**0.0**	2.20
*J. Theor. Biol.*	2048	0.8	100.0	100.0	100.0	23.6	**23.7**	**12.5**	1.55

The table gives first the total number of articles examined in each journal, and then the percentages of articles dealing with soil ecology (Total soil), based on modelling (Model), or based on an evolutionary approach (Evolution). In these two latest cases three percentages have been distinguished: total percentages of articles (Total), percentages of articles based on a model or an evolutionary approach within non soil ecology articles (Within non soil) and percentages of articles absed on a model or an evolutionary approach within soil ecology articles (Within soil). Asterisks in the “Model-Within soil” and “Evolution-Within Soil” columns denote a significant difference (χ^2^ test, df = 1, P<0.05) between the percentages among all non-soil ecology articles (Within non soil) and soil ecology articles (Within soil). Bold characters denote cases for which a journal publishes more model- or evolution-related articles within its non-soil articles than within its soil articles. Dotes in the “Model-Within non soil” and “Evolution-Within non soil” columns denote the fact that soil ecology journals only publish articles related to soil ecology. Impact Factors (IF) are given for 2003.

At the same time, generalist journals publish much more studies using modelling (between 11.4 and 36.2%) than soil ecology journals do (between 0.4 and 6.3 %, see Table 1). It must be marked that the two generalist journals publishing the less model-based studies are the ones publishing the more soil-related papers (*Functional Ecology* and *Journal of Ecology*). Conversely, ecology journals specialized in modelling (*Theoretical Population Biology* and *Journal of Theoretical Biology*) publish few papers about soil ecology (respectively 0.8 and 1.3 %). *Ecological Modelling* which is less theoretically oriented publishes a higher percentage of papers dealings with soil (10.7 %). There is a significant negative correlation between the proportion of papers dealing with soil ecology and the proportion of papers based on models (Fig. 1).

In the same vein, generalist papers publish much more studies dealing with evolution (between 10.6 and 45.6 % in most cases, 10.6 for *Oikos*, but 3.9 % for *Journal of Ecology*) than journals specialized in soil ecology (between 0.1 and 1 %, see Table 1). Again, the generalist journal publishing the highest percentage of soil ecology-related articles, *Journal of Ecology*, also publishes the fewest studies dealing with evolution. Overall there is a significant negative relationship between the percentage of articles dealing with soil ecology and the percentage of articles tackling evolutionary issues (Fig. 1).

We also tested whether evolutionary journals publish high percentages of studies using modelling and conversely that theoretical-oriented journals publish high percentages of studies dealing with evolution. These hypotheses hold for all journals but for *Ecological Modelling* whose papers rarely deal with evolution (Tab. 1). This journal publishes more papers about soil ecology than *Journal of Theoretical Biology* and *Theoretical Population biology*. This suggests the existence of a link between evolutionary thinking and ecological modelling and confirms the independence of soil ecology from this evolutionary-modelling pole.

In most cases, in journals non-specialized in soil ecology, the percentage of articles using a model or an evolutionary interpretation is lower for soil ecology articles than for the other articles and most of these differences were significant (Table 1, χ^2^ tests). The exceptions mainly correspond to journals publishing low numbers of articles dealing with soil ecology (*American Naturalist*, *Evolutionary Ecology*, *J. Animal Ecology*). In these cases, very few articles (fewer than 10) are concerned so that the validity and significance of χ^2^ tests are dubious. Overall, when journals non-specialised in soil ecology or theory are pooled, respectively 11.4 % and 20.2 % of soil ecology articles and non-soil ecology articles use modelling. This difference is highly significant (χ^2^ test, df = 1, P<0.001). Similarly, in journals non-specialised in soil ecology or evolution, respectively 7.3 % and 29.4 % of soil ecology articles and non-soil ecology articles use evolutionary interpretations. This difference is also highly significant (χ^2^ test, df = 1, P<0.001). These results suggest that soil ecologists use more parsimoniously modelling and evolutionary approaches than ecologists of other fields.

*Science* and *Nature* publish both about 0.4 % of papers having a connection with soils. Among these articles, about 40 % (41.96 % for *Nature*, 45.56 % for *Science*) of the published studies deal with purely physical issues such as transport of particles at a global scale, and with mars and lunar soils. It remains about 60 % of terrestrial soil-related articles (about 0.25 % of all published articles) that can be considered as dealing with soil ecology (see above explanations on the type of studies considered as soil ecology). For *Nature* and *Science*, respectively, 50 % and 25 % of these studies related to soil ecology deal with global change issues. Typically, the response of a soil parameter or a soil community to an increase in the atmospheric CO_2_ level or temperature is examined [10], [11]. Such studies are of course important in the present context. However, many of them tend not to analyse directly the specific and poorly known mechanisms linking soil microflora, soil macroorganims, plants and soil processes [but see12,13].

**Figure 2:**
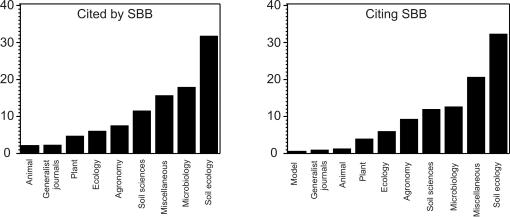
Citation practices in soil ecology. Distribution of articles cited by *Soil Biology & Biochemistry* (left panel) and citing articles published in Soil Biology and Biochemistry (right panel) according to the category of journal they have been published in. Issues 10, 11 and 12 of the volume 35 (2003) of *Soil Biology & Biochemsitry* have been scanned in 2007 using the bibliographic data base ISI Web of Science, so that article citing these issues of *Soil Biology & Biochemistry* have been published between 2003 and 2007 while all the articles cited in these issues have been taken into account whatever their year of publication. Categories of journals are described in the text.

Figure 2 displays the distribution of articles cited by and citing the articles of the three sampled issues of SBB. 43 articles have been published in these issues. They cite about 1400 other articles and have so far been cited by about 300 articles. These articles (citing SBB and cited by SBB) have nearly the same structure according to our classification. About 6 % of these articles belongs to the category “General ecology”. No journal specialized in evolutionary ecology cites SBB or is cited by SBB. None of the articles cited by SBB and only two articles citing SBB have been published in journals specialized in modelling. The majority of articles cited by or citing SBB have been published in soil ecology journals (about 30%). About 18 and 12% of these articles have been published in microbiology journals. About 10 % of these articles cited have been published in journals specialized respectively in soil sciences or agronomy.

## Discussion

### 1 Links between soil ecology, general ecology, modelling and evolutionary approaches

There are low percentages of cross-citations between soil ecology journals and journals specialized in modelling and evolutionary approaches. Moreover, soil ecology journals publish low percentages of articles based on modelling and evolutionary approaches and, conversely, journals specialized in evolutionary and modelling approaches publish low percentages of soil ecology-related articles. Even inside the journals non-specialized in soil ecology, articles related to soil ecology use less often models and evolutionary approaches than the other articles published in the same journals. Although this does not replace a much wider bibliographic study comparing different fields of ecology (soil ecology, marine ecology, bird ecology … etc), this suggests that soil ecology uses modelling and evolutionary thinking more parsimoniously than other fields of ecology. Besides, soil ecology is not well represented in generalist ecology journals. Finally, SBB (and probably other soil ecology journals) is little cited by or does not cite much generalist ecology journals, theoretical ecology journals and evolutionary ecology journals, while cross-citation could be a way to make up for the lack of modelling and evolutionary orientated articles published in soil ecology journals. Reciprocally, we could have expected generalist journals to cite more often specialized journals such as soil ecology journals as a source of patterns to be interpreted and data to test their general theories. All these results confirm the relative independence between soil ecology and general ecology and the five hypotheses presented in the introduction.

It must be marked that the trend described using journal articles is confirmed by the examination of text books on soil ecology that hardly mention explicitly evolutionary issues and model results [14], [15]. Meanwhile, at least some text books on plant ecology [16], [17] and insect ecology [18], [19] or aquatic ecosystems [20], [21] largely refer to the advances made using models and evolutionary thinking.

In the following discussions, we try to interpret these results and more particularly the relative independence between soil ecology and general ecology, and the parsimonious use of modelling and evolutionary interpretations made by soil ecology. We first propose two explanations that could explain some of our results and show that they are not valid or do not explain the whole bibliographic pattern we have described. A first explanation would be that soil ecologists have had to face more technical problems than other ecologists (section 2). That would have impeded them to develop their field as fast as in other fields of ecology and would have slowed down efforts of modelling and evolutionary questioning. We argue that this explanation contains some truth but is not sufficient to explain the whole bibliographic pattern we have described. A second explanation would be that generalist journals, with higher impact factors, necessarily publish higher percentages of model- or evolutionary-based studies because modelling and evolutionary approaches lead to more general results (section 3). We show that this explanation does not hold. We then propose that the scarcity of modelling and evolutionary approaches in soil ecology is an important proximal cause of the relative independence of soil ecology from general ecology (section 4). In this section we thus detail the reasons while soil ecology would highly benefit from more conceptualisation and evolutionary thinking. We then try to propose some historical reasons for the whole bibliographic pattern we have described and particularly for the scarcity of evolutionary and modelling approaches in soil ecology (section 5). We finally conclude by some remarks on the role of the publication system on the development of soil ecology and suggest that this system might hamper interdisciplinary thinking and the building of links between general theories and specific empirical studies (section 6).

### 2 Technical difficulties, so what?

A first explanation for the relative independence between soil ecology and general ecology would be that soil ecology is intrinsically difficult to study due to the following points: (1) Soil is a black box. It is more difficult to manipulate and observe soilo rganisms without disturbing their environment than above-ground organisms. (2) Soil is a very complex environment in which it is difficult to disentangle biotic and non-biotic interactions. (3) These interactions involve a solid phase, an aquatic phase, a gaseous phase and complex exchanges between these phases. (4) Soils are extremely heterogeneous at all special scales [from the micron to the kilometre, 22]. (5) Soil processes depend directly on a huge variety of organisms, whose size is typically low and whose taxonomy and diversity are poorly known in comparison to aboveground organisms. (6) Soil processes involve a huge variety of organic molecules and chemical reorganizations that are poorly understood. Possibly, soil ecologists have, for the moment, focussed on solving their technical problems and developing methods to investigate soil processes. This could contribute to explain that they are centred on their own discipline and less open to general ecology. For example, the development of investigation methods has involved the development of molecular techniques in soil microbiology.

The technical difficulties could thus partially explain why soil ecology makes a parsimonious use of models and evolutionary rationales and why soil ecology has developed partially independently of general ecology. However, these difficulties have not impeded the production of a huge empirical corpus and technical problems are progressively overcome by new methods based, for example, on molecular biology [23], or stable isotope marking [24]. Besides, a huge number of soil ecology articles use already validated methods and standard protocols. This suggests that technical difficulties are not the only reason why soil ecologists parsimoniously use models and evolutionary interpretations.

### 3 Generalist journals should not publish more model and evolutionary oriented articles than specialized journals

To explain that generalist journals publish more studies based on modelling than soil ecology journals, it could be simply argued that, by definition, generalist journals with high IF publish studies addressed to a wider readership than specialized journals and that modelling help obtaining general arguments that are likely to attract a wide readership. This is probably true (see below) but does not explain the whole bibliographic pattern. First, we have also found that, inside journals not-specialized in soil ecology, soil ecology articles are less based on modelling and evolutionary approaches than the articles of other field of ecology. Second, this explanation assimilates implicitly models to theoretical models that indeed aim at testing and building general theories. In fact, they are many kinds of models that can be classified according to the degree of generality, realism and precision of the predictions [25]. To be general a model must take into account few fundamental mechanisms but remains a non-fully realistic idealization and cannot give quantitatively precise predictions because it has not been fitted to any particular system. Conversely, such predictions require taking into account more mechanisms and more particularities of the modelled ecological systems so that models giving precise predictions cannot be general [25]. Recent analyses of Levin's classical article support the existence of tradeoffs between the different type of models he has described [26], [27].

Clearly, building general theoretical models is useful but it is also imperative to build models focussing on particular systems. We thus suggest that journals specialized in soil ecology should publish more studies based on models that apply to soil systems. These studies should encompass theoretical models applying to soil systems in general (to build general theories on soil ecology, e.g. a general model showing the implications for decomposition of the existence of two pools of organic matter with a different degree of recalcitrance) or models built to answer specific questions on particular soil systems (a model that predict the decomposition rate of soil organic matter in a given site as a function of climatic variations). Meanwhile, generalist journals could publish a theoretical model studying the effect of decomposition rate on primary production if its conclusion can be applied to a wide class of systems (for example both on soil and aquatic systems). We want to emphasize that it is only by applying different modelling approaches to study the same issue that the robustness [25] and relevance of modelling results can be assessed. In particular, while general and theoretical models are efficient to suggest broad theories to be tested, more realistic and precise models are more efficient to compare model outputs to empirical observations.

The low percentage of articles linked to evolutionary issues published in soil ecology journals can be interpreted in the same way as the low percentage of articles using models published in these journals: evolutionary interpretations help to reach general conclusions that could be applied to many systems. The parallel drawn here between the way models and evolutionary interpretations can help building general theories is confirmed by the high percentages of models published in journals specialized in evolution and the high percentages of evolution-related studies published in journals specialized in modelling (with the exception of Ecological Modelling). Indeed, some evolutionary mechanisms and issues are very general and results about them should be published in generalist journals (especially when they are studied using theoretical models). However, other evolutionary mechanisms and issues only concern particular ecological systems. Once again, soil ecology journals should publish articles involving evolutionary rationales that are particular to soil systems or studying how general evolutionary questions translate into soil ecology. For example, generalist journals should publish general results on the way dispersal ability and other life-history traits coevolve while soil ecology journals should publish studies on the consequences of the limited dispersal ability of many soil organisms on the evolution of their life-history.

### 4 Proximal explanation of the relative independent development of soil ecology and general ecology

An explanation would be that soil ecologists tend to present their results in such a way that they are poorly linked to general theories of ecology, i.e. they rarely interpret their results in the lights of widely accepted ecological theories such as food-web, competition, coexistence or evolutionary theories. Their articles would thus be more difficult to publish in generalist journals and would be mostly cited by other soil ecologists (as confirmed by our analysis on SBB), which would at last result in lower numbers of citations and specialized journal with low IF. According to this explanation the lack of modelling and evolutionary interpretation would be one of the causes of the weak connection between general ecology and soil ecology. We detail below how soil ecology would benefit from an effort of conceptualisation and evolutionary thinking.

Modelling is an integral part of natural sciences in the sense that conceptual verbal models are already models. Such models are necessary to sort out hypotheses and make clear statements about our mental representation of nature and the links between observed patterns and mechanisms [28], [29]. Then, mathematical models are necessary when the consequences of hypothesized processes cannot be predicted verbally which arises as soon as the studied systems become complex (more compartments, more interactions, retroactions, space is explicitly taken into account). Moreover, without prejudging the existence of general laws in ecology [30], [31], conceptual verbal models and results of mathematical models sum up our ecological knowledge. The model results that are widely accepted at a given time often constitute a paradigm in the Kuhnian acceptation [32]. Taken together, models, either mathematical or verbal, are necessary both to predict the consequences of newly discovered processes and to provide new hypotheses to be tested empirically.

In this way, soil ecologists should more often take advantage of modelling results generated in other fields of ecology to build new hypotheses suggesting in turn new experiments. Conversely, they should benefit from constructing more often their own models to explain their empirical results. An effort of conceptualisation should help soil ecology to build its own paradigm and thus to become more visible and independent. It should also help soil ecology to build more bridges with other fields of ecology because models are easier to compare than experimental data gathered on very different systems. These two trends, independence and integration, are both necessary to foster progress in a given scientific field [33], [34].

An effort of conceptualisation in soil ecology will also lead to the development of new theories on aspects of ecology often overlooked by general ecology. For example, relations between soil organisms often involve ecosystem engineering activities [35], exchanges of signal molecules [see for example 36], and relations mediated through the recycling of nutrients [37], [38]. Such relations have so far been poorly taken into account by classical food web models [39]. Their consequences on population dynamics, community stability and evolution are poorly understood. Moreover, many soil processes are based on the activity of microbes. Linking the population dynamics of microbes, their plasticity and their capacity to evolve to soil and whole ecosystem properties is a new field of investigation that requires new models linking population dynamics, functional ecology and evolution [40]–[42]. We of course do not wish to suggest that there has not been any effort of conceptualisation in soil ecology. For example, Clarholm's work on the microbial loop [43], without the help of any mathematical model, has a long lasting influence on soil ecology researches. Setälä's work on decomposer biodiversity [44] is also based on a strong conceptual base and many other good examples can be found [see for example in 45]. However, we think that these efforts of conceptualisation should be reinforced and become a habit of all soil ecologists.

Although describing the proximate mechanisms involved in the dynamics of populations and ecosystems is a challenging task, it is primordial to take into account the fact that organisms are the result of a long evolution process. The biological traits that determine the nature of interactions between organisms have also evolved. As models, evolutionary arguments can interplay bidirectionally with empirical results, helping to interpret empirical results and suggesting new experiments. Thus, besides studying evolution for its own sake, studying the evolution of soil organisms should be scientifically beneficial for three reasons. (i) Interpretation of empirical results must be consistent with evolutionary knowledge. For example, when new ecological interactions or new biological traits are pointed out, their ecological significance must take into account the fact that these traits have evolved and thus that they should benefit to their owners or that their evolution is linked to a constraint. It is relevant to identify the nature of this benefit or constraint. (ii) Conversely, evolutionary theories and new evolutionary models applied to soil issues are likely to lead to predictions on the way biological organisms have evolved and thus on the biological traits of present soil organisms and the type of interactions that link them. Such predictions can be used as guides to design new experiments. (iii) Finally, it is more and more recognized that evolution is often quicker than formerly believed [46], and thus that some temporal patterns observed on the human time scale could be due to evolutionary processes. This should particularly be the case for soil processes because they depend on short-lived organisms with a high potential for rapid adaptation such as bacteria or protozoa [6], [40].

All these explanations suggest that developing a theoretical and evolutionary framework for soil ecology should benefit grandly soil ecology and ecology in general as already mentioned [7]–[9]. What are the historical reasons of the absence of such a framework and the scarcity of links between soil ecology and general ecology?

### 5 Historical explanations

Soil ecology is historically much more based on the ecosystem paradigm of ecology than on its population paradigm [34]. One reason might be linked to the close dependence of soil organisms on chemical and physical constraints [14] and the importance of physical and chemical processes in soils. This is confirmed by a study on trend words in ecological journals which shows that “below-ground” is associated to the abiotic pole of ecology [47]. This is also confirmed by the quantitative importance of articles published in journals emphasizing chemical and physical soil processes among the articles cited by and citing SBB (Fig. 2). The close link between ecosystem ecology and soil ecology can explain partially the poor use made by soil ecologists of mathematical models and evolutionary knowledge since population ecology has always been more theory- and evolution-orientated. In the same vein, soil ecology is historically linked to agronomy [48] as confirmed by the high number of citations between SBB and agronomy journals (Fig. 2). This contributes to explain the relative independent development of general ecology and soil ecology [48], [49]. Moreover, agronomy has so far mostly aimed at gains in production and is consequently poorly evolution-oriented.

### 6 Partial responsibility of the publication system

We finally acknowledge the fact that our approach, i.e. studying scientific thinking through counts of articles published in different journals using very broad thematic categories, is slightly naïve and simplistic. However, all scientists know that choosing the journal to submit a manuscript is of paramount importance to get published and to get a wide readership. Whether we want it or not, in an academic scientific world where we have to “publish or perish”, journals influence greatly the way we work and probably the way we think. We suggest three general drawbacks of the classification of journals according to their degree of generality and their Impact Factor.

First, this classification might deter ecologists who are specialists of a given field of ecology from reading and quoting more theoretical works or results from other fields of ecology. Conversely, it might deter more generalist ecologists and theoreticians to read and quote more specialized studies. This classification opposes “particularity” and “generality” while our goal, as scientists, should be to derive as many links as possible between these two poles [34]. Of course, generalist journals could be the place where “particularity” and “generality” should be confronted but we have shown that it does not work efficiently, at least in soil ecology. Of course, many bibliographic databases are generalist and interrogating them with a given keyword yields articles published in many journals whatever their degree of generality and their Impact Factor. However, the superabundance of published papers impeding to read exhaustively the relevant literature, the temptation is great to focus on a handful of journals. For example, soil ecologists might already have difficulties to read all relevant articles published in specialized journals and might not have the time to read articles published in other journals. If, by chance, they do so they are not likely to focus on theoretical and evolutionary articles because it is not in the “style” of the specialized journals to which they intend to submit their own work to quote such studies.

Second, we know that to publish in a journal it is important to submit to the “style of the journal”. Journals delimit their respective scopes and styles (through intentional choices, self-organization and competition) so that it might be difficult to publish articles that do not fall within one of the categories journals have created. It might for example be difficult to publish models in soil ecology journals or soil ecology studies in generalist journals because soil ecology has historically developed partially independently from general ecology and theory. In other words, scientific structuring by traditional journals is likely to slow down changes in the structure of sciences and limit interdisciplinary studies. This might be particularly detrimental for the development of ecology which is by nature interdisciplinary because it aims at linking (1) the physical and biological worlds, (2) natural and human sciences (because mankind is one of the most influential component of the biosphere and to develop applications), (3) many different scales (from genes and molecules to atmospheric circulation).

Third, the classification of journals according to a gradient of generality, as denoted by their Impact Factors, and the fact that these Impact Factors are often considered as an index of intrinsic scientific quality might be counterproductive. The Impact Factor of a journal has been demonstrated to be a poor predictor of the number of citations an article will finally get [50]. Indeed, reviewers and editors are able to control the scientific soundness of a study, the relevance of the protocols and the consistency between results and conclusions. It is much more difficult to predict a priori the level of generality of the study and its real usefulness.

Taken together, our bibliographic study and these last paragraphs emphasize the utility of generalist interdisciplinary journals, such as PLoS ONE, that select their articles on a technical base and not because they fit to the journal style, approach, subject and supposed level of generality. Such journals are likely to follow scientific progresses in a quick and flexible way and foster more connections between disciplines. In such a publication system articles will no longer be ranked according to the Impact Factor of their journals but according to the number of citations they really get. Articles will thus be ranked according to their “usefulness” and not according to an a priori level of generality. We finally think that the development of this type of journal should be especially profitable for soil ecology that has developed, as we have shown, too independently from the rest of ecology, theoretical ecology and evolutionary ecology.
